# 

**Published:** 1893-07

**Authors:** 


					﻿CONTENTS*
PAGE.
Popular Errors in Medicine 145
Epilepsy.....<......... 148
What is Cholera? ....... 150
Family Order........... 154
Medicine Taking........ 156
Oranges as Medicine..... 158
PAGE.
Night Air. .*............ 159
Milk instead of Medicine. 160
Against Pungent Odors... 161
Editor’s Outlook......... 162
Literary................. 164
Recreation Bureau Noles. 166
				

